# State-of-the-art sleep arousal detection evaluated on a comprehensive clinical dataset

**DOI:** 10.1038/s41598-024-67022-9

**Published:** 2024-07-14

**Authors:** Franz Ehrlich, Tony Sehr, Moritz Brandt, Martin Schmidt, Hagen Malberg, Martin Sedlmayr, Miriam Goldammer

**Affiliations:** 1 Faculty of Medicine and University Hospital Carl Gustav Carus, TUD Dresden University of Technology, Dresden, Germany; 2 Department of Neurology, University Hospital Carl Gustav Carus, TUD Dresden University of Technology, Dresden, Germany; 3 Institute of Biomedical Engineering, TUD Dresden University of Technology, Dresden, Germany

**Keywords:** Sleep, Sleep disorders

## Abstract

Aiming to apply automatic arousal detection to support sleep laboratories, we evaluated an optimized, state-of-the-art approach using data from daily work in our university hospital sleep laboratory. Therefore, a machine learning algorithm was trained and evaluated on 3423 polysomnograms of people with various sleep disorders. The model architecture is a U-net that accepts 50 Hz signals as input. We compared this algorithm with models trained on publicly available datasets, and evaluated these models using our clinical dataset, particularly with regard to the effects of different sleep disorders. In an effort to evaluate clinical relevance, we designed a metric based on the error of the predicted arousal index. Our models achieve an area under the precision recall curve (AUPRC) of up to 0.83 and F1 scores of up to 0.81. The model trained on our data showed no age or gender bias and no significant negative effect regarding sleep disorders on model performance compared to healthy sleep. In contrast, models trained on public datasets showed a small to moderate negative effect (calculated using Cohen's d) of sleep disorders on model performance. Therefore, we conclude that state-of-the-art arousal detection on our clinical data is possible with our model architecture. Thus, our results support the general recommendation to use a clinical dataset for training if the model is to be applied to clinical data.

## Introduction

### Sleep and arousal

Good quality and quantity of sleep have major impact on health and overall quality of life^1^. One substantial measure of sleep quality are sleep arousals, as they provide deep insights into the pathophysiology of sleep disorders and sleep quality^2^. In general, arousals are short transient wakening reactions that can lead to a more fractured sleep^3^, but they are also an important part of the normal sleep process^2^.

Scoring of arousals is part of the standard diagnostic procedure for sleep analysis. The American Academy of Sleep Medicine^4^ has defined the standard process for annotating arousals. Arousals are detected visually by an expert. This is done using the electroencephalogram (EEG) and electromyogram (EMG), which are part of polysomnography (PSG). According to the American Academy of Sleep Medicine (AASM), an arousal is an abrupt shift in the frequency of the EEG that lasts at least three seconds and requires ten seconds of preceding sleep^4^. In practice, this results in a maximum length of 15 s, otherwise the epoch would be scored as a wake epoch. During the stage of rapid eye movement sleep (stage R sleep), scoring an arousal also requires a simultaneous increase in the submental EMG^4^. Arousals are spontaneous or may occur in response to sleep-disrupting events such as apneas, hypopneas, respiratory effort-related arousals (RERAs), and periodic leg movements. Arousal detection is a time-consuming process and requires a trained expert. Scoring is also influenced by individual raters, resulting in lower inter-rater and intra-rater reliability^5^, and may be biased by the knowledge of the specific diagnosis. In conclusion, manual scoring has two disadvantages: (1) it is tedious and time-consuming, even for experts, and (2) the results might be subjective.

### Challenges in automation and machine learning

Automated arousal detection is therefore of great interest. It promises to be fast, objective and reliable. However, the complexity of the underlying pathophysiological processes and how they affect the biosignal has made automatic arousal detection a challenge. According to Qian et al.^6^, there are several approaches that try to solve this problem using signal processing, traditional machine learning with feature extraction and deep neural networks. The latter has shown the most promising results on large datasets in recent years^6^. However, there are three major limitations with current approaches to arousal detection:

- lack of patients or patient diversity in the test sets
- there is no medical evaluation of how well the models would perform in different patient groups
- the metrics used do not provide information on relevant clinical parameters

The first two limitations are straightforward: a good test set should represent the range of patients who visit a sleep laboratory. Therefore, it should include patients of different ages, genders and sleep disorders. The lack of such diversity in current publicly available datasets limits the real-world applicability of automated arousal detection models. Additionally, a certain number of patients in the test set is required for statistical purposes. Furthermore, medical evaluation requires medical information such as sleep disorder diagnosis.

In contrast, the third problem is more specific to the setting of an automated detection. Machine learning requires segmentation of a signal to predict events, and these segments can then be evaluated with metrics against the ground truth. However, because they are applied to segments of fixed size, these metrics do not provide information about the actual arousal events. According to the AASM, the number of arousals and the arousal index (ArI: number of arousals during one hour of sleep) are the only parameters to be reported for PSG that are solely influenced by arousal detection^4^. A common evaluation strategy might be to consider only 30 s sleep epochs^7,8^ and compare whether there is any arousal at all in that epoch. However, these strategies may not translate well into clinical parameters. Other approaches use a very small segment size of 5 milliseconds^9,10^, which consequently requires a strategy to merge the segments into events.

### Related work

Attempts to automate arousal detection date back to 1998^11^, where a feed-forward network was used to analyse the EEG, EMG and EOG signal of eight subjects to determine sleep stages and the k-mean method to determine sleep arousals. In a following study^12^, two EEG and one EMG signal were used from eleven patients with sleep disorders to automatically detect arousals by using wavelet transformation. Both experiments showed promising results for automating arousal detection and laid the foundation for further research. Interest accelerated in the following years with the publication of the importance of arousals during sleep in 2004^2^. In 2005 Cho et al.^13^ used time–frequency analysis and a support vector machine classifier on a single EEG from nine patients to detect arousals. The EMG and two EEGs of 20 patients were used by Alvarez-Estévez and Moret-Bonillo^14^ to detect arousals using different signal processing steps.

Research reached a peak with the public availability of large (n > 1000) datasets^15–17^ and the PhysioNet challenge in 2018^18^. The PhysioNet challenge presented a dataset of patients monitored in a sleep laboratory to diagnose sleep disorders. The approaches from Howe-Patterson, Pourbabaee, and Benard^9^ and Li and Guan^10^ achieved the best results in the challenge using machine learning. Using the test set of 989 patients, they were able to make better assumptions about the generalisability of their models. The approach of Li and Guan^10^ even showed the portability of their model architecture to the SHHS dataset. Unfortunately, the PhysioNet challenge did not provide information about the patients’ medical conditions and diagnoses, so medical evaluation was still not possible, and the approaches only used the PhysioNet challenge scoring for comparison, which does not include information about the number of arousals.

In Olesen et al.^19^ applied machine learning to detect arousals and leg movements during sleep using a test set of 1000 male participants. They improved this approach in 2020^20^ by using different setups for their EEG channels, and achieved similar results on the test set with only a third of the training data. This approach was later extended by Zahid et al.^21^ by combining arousal detection with leg movement and sleep-disordered breathing, presumably using the same test set of 1000 male participants. They also included the correlation between the calculated ArI and the manually scored ArI in their evaluation.

The approach of Alvarez-Estevez and Fernández-Varela^7^ uses a more diverse dataset with a test set of 2296 participants from the SHHS study combined with 472 patients from their clinical sleep laboratory. This is also one of the few attempts to include the effect on ArI.

A comparison of the different metrics used in more recent approaches can be found in Table 1.

**Table 1:** Results compared with the literature.

Approach	Signals	Year	Database	Test Set	Resolution	AUPRC	F1*	ArI-Diff Mean ± Std	ArI-Diff Median	ArI r	ArI error Q2 (Q1–Q3)
Howe-patterson, pourbabaee, and benard^9^	Multiple	2018	PhysioNet	989	5 ms	0.54					
Olesen et al.^19^	EEG, EOG, EMG	2019	MrOS	1000	multiple		0.75				
Alvarez-estevez and fernández-varela^7^	EEG, EMG	2019	SHHS2	2296	30 s		0.59	+ 0.35 ± 4.89	0.44	0.78	
			HMC-M	252			0.57	-0.52 ± 6.68	0.6	0.79	
			HMC-S	220			0.64	+ 0.84 ± 5.41	0.40	0.77	
Brink-kjaer et al.^27^	EEG, EOG, EMG	2020	MrOS, CFS, WSC, SSC	1026	1 s		0.76				
			MrOS	580			0.79				
			CFS	145			0.75				
			WSC	271			0.70				
Li and guan^10^	Multiple	2021	PhysioNet	989	5 ms	0.55					
			SHHS1	1000		0.59					
			SHHS2	1000		0.60					
Zahid et al.^21^	EEG, EOG, EMG	2023	MrOS	1000	multiple		0.70			0.85	
Our approach	EEG, EOG, EMG	2024	SHHS1	1698	20 ms/1 s	**0.83**	0.80	**0.05 ± 3.58**	**0.29**	0.88	**1.71 (0.75–3.27)**
			MESA	616		0.82	0.81	2.34 ± 6.91	1.58	0.91	2.86 (1.33–5.10)
			MrOs	815		0.82	0.80	1.47 ± 7.37	1.77	**0.95**	2.29 (0.99–4.57)
			DSDS	1202		0.71	0.74	1.53 ± 9.17	1.68	0.78	2.18 (1.01–4.20)

*The different F1 values are not comparable due to different resolutions and label manipulation. ArC (Arousal Count), ArI (Arosual Index), r (Pearson correlation).

Bold values indicate the best value for each metric.

### Our approach

We addressed the current limitations using a large dataset from our clinical sleep laboratory, at the *University Hospital Carl Gustav Carus in Dresden*. The dataset includes records from over twelve years of daily work. This allowed us to evaluate our algorithm on a wide variety of patients and to assess the influence of different sleep disorders, age groups and sex on the performance of our model. We also used an event-based evaluation to determine the quality of arousal detection and calculated the ArI for each record to determine the impact on diagnosis. We also validated the portability of our approach to different datasets and how a model trained on these datasets would perform in our sleep laboratory. Our aim was to achieve state-of-the-art results with a machine learning algorithm trained on clinical data from the daily work of a sleep laboratory and to investigate the impact of sleep disorders on publicly trained models. To the best of our knowledge, there is no published approach using a dataset of this size from day-to-day work in a sleep laboratory, nor an evaluation of the effect of different sleep disorders on an automated sleep arousal detector.

## Methods

### Datasets

A successful deep learning approach requires a heterogeneous dataset with a large diversity of patients and enough labels to learn from. For model development and optimization, we used our own clinical Dresden Sleep Dataset (abbreviated as “DSDS” in the following text), which allows us to evaluate over additional clinical data. For reasons of reproducibility and to investigate the portability of our approach, we used equally large datasets that have been made publicly available on the National Sleep Research Resource (NSRR)^22^. An overview of the datasets can be found in Table 2.

**Table 2:** Characteristics of our own Dresden Sleep Dataset (DSDS), first part of the Sleep Heart Health Study (SHHS1)^15^, Multi-Ethnic Study of Atherosclerosis (MESA)^17^ and MrOS Sleep Study (MrOS)^16^.

	DSDS	MESA	SHHS1	MrOS
General information				
Full night PSGs	3423	2056	5793	3933
Patients/Participants #	1755	2056	5793	3933
Age	57.75 ± 15.78	69.35 ± 9.08	63.14 ± 11.23	77.70 ± 5.60
% Female	40.29	53.60	52.36	0
BMI (kg/m^2^)	–	–	28.16 ± 5.09	26.95 ± 3.82
% nights with therapy devices	40%	0%	0%	0%
Sleep information				
Total sleep time (min)	369.12 ± 83.18	359.77 ± 82.60	359.90 ± 64.49	346.24 ± 70.45
Sleep latency (min)	16.17 ± 24.35	45.43 ± 41.40	14.08 ± 20.41	24.66 ± 26.94
REM latency (min)	139.60 ± 89.33	108.88 ± 76.24	73.73 ± 44.38	93.18 ± 66.75
WASO (min)	75.50 ± 56.00	94.49 ± 65.36	61.46 ± 44.02	106.76 ± 59.58
Sleep efficency (%)	78.70 ± 15.08	75.74 ± 13.46	82.77 ± 10.54	76.75 ± 11.81
Arousal count	162.63 ± 97.16	129.41 ± 71.00	110.49 ± 64.35	135.23 ± 68.27
Arousal index (/h)	26.88 ± 15.84	22.32 ± 12.06	19.16 ± 10.66	23.93 ± 12.37
periodic limb movements (PLM) index (/h)	35.68 ± 41.91	14.22 ± 24.99	–	36.59 ± 37.11
PLM index with arousal (/h)	5.14 ± 7.53	1.56 ± 3.57	–	4.22 ± 5.82
Apnea–hypopnea index (/h)	16.04 ± 19.32	20.07 ± 17.75	14.46 ± 14.48	17.64 ± 15.46
Diagnosis				
Normal findings	8%			
Sleep-related breathing disorders	73%			
Sleep-related movement disorders	17%			
Insomnia disorders	16%			
Hypersomnia disorders	5%			
Parasomnia disorders	13%			

Normal findings: No evidence of sleep disorder or only mild Sleep-related breathing disorders.

Sleep-related breathing disorders: includes obstructive sleep apnea (OSA), central sleep apnea, complex OSA, Hypoventilation.

Sleep-related movement disorders: includes restless legs syndrome, periodic leg movement disorder, other sleep-related movement disorders.

Hypersomnia disorders: includes narcolepsy type 1/type 2, Kleine-Levin syndrome, chronic insomnia disorder.

Parasomnia disorders: includes REM sleep behaviour disorder (RBD), Non rapid eye movement (NREM) parasomnias.

#### DSDS

The DSDS dataset was recorded in the sleep laboratory of the Department for Neurology at the *University Hospital Carl Gustav Carus in Dresden*. This interdisciplinary sleep laboratory examines and treats patients from the entire spectrum of sleep medicine. The focus is on the diagnosis and treatment of neurological sleep disorders: parasomnia, hypersomnia (narcolepsy) and restless legs syndrome. Patients were referred to the sleep laboratory by their general practitioner or specialist. Therefore, they all they all had specific sleep disorders or self-reported sleep problems. The dataset was collected retrospectively during the European Regional Development Fund project “Tele-Schlaf-Medizin”. It contains 7677 PSGs for 3125 patients from 2008 to 2020. The use of the data and methods for this research was done in accordance with relevant guidelines and regulations, and approved by the ethics committee of TUD Dresden University of Technology (BO-EK-92032020), which allowed the retrospective use of the pseudonymized data without informed consent.

PSGs were recorded using the Philips Alice 5 diagnostic system. Most PSGs were recorded in a standard setup according to AASM criteria, including.

- six-channel EEG (F3:A2, F4:A1, C3:A2, C4:A1, O1:A2, O2:A1, A1:A2)
- two-channel electrooculogram (EOG),
- two-channel chin EMG,
- leg EMGs,
- nasal pressure and (in later recordings) thermistor signal as airflow signals,
- chest and abdomen belts as respiratory effort signals,
- oxygen saturation by finger oximetry,
- body position sensor,
- electrocardiogram,
- additional snoring microphone,
- synchronized PSG video.

The signals, including all annotations, were stored electronically in a proprietary data format. Export to machine-readable formats was performed using the Alice 6 PSG diagnostic system.

**Figure 1:**
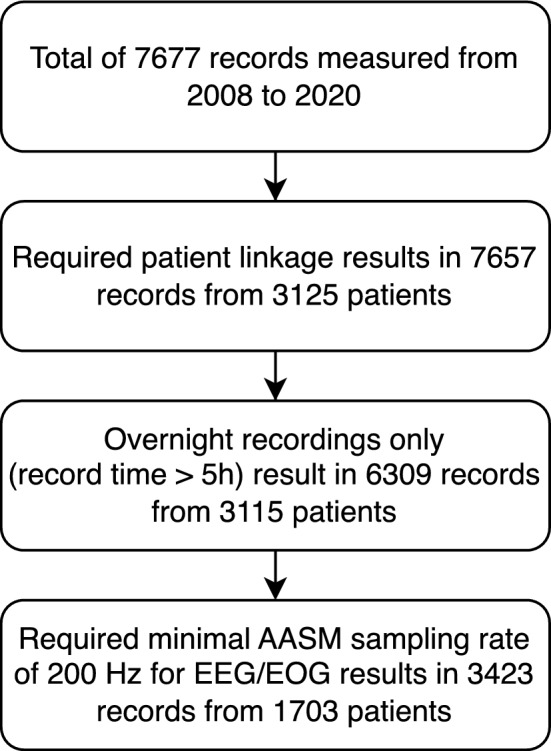
Procedure on selecting relevant recordings for the DSDS.

To separate the dataset by patient, we excluded records that could not be associated with a patient, resulting in 7657 records from 3125 patients. As we wanted to detect sleep arousals, we only included overnight PSG recordings with a minimum record duration of 5 h. This resulted in 3115 patients with a total of 6309 PSG recordings. We excluded recordings with an EEG sampling rate of less than 200 Hz, as recommended by the AASM and as necessary to reproduce state of the art results, e.g. by Li et al.^10^. The final dataset contains 1703 patients with a total of 3423 PSG recordings, recorded from 02/2008 to 11/2020. The record selection procedure is shown in Fig. 1.

The dataset has a heterogeneous patient population ranging in age from 18 to 92 years and 40% are women. The average ArI is 27/h and the average Apnea–hypopnea index (AHI) is 16/h. The average sleep duration is 369 min. Most of the patients spent two consecutive nights in the sleep laboratory. In the majority of the cases one diagnostic night and one night with therapy were recorded, but two consecutive diagnostic nights were common as well. The dataset includes information on whether a patient was treated for sleep apnea, which accounts for a total of 40% of nights.

Recordings were scored by trained and experienced PSG technologists and somnologists according to AASM guidelines. Sleep disorder diagnoses were based on the International Classification of Sleep Disorders^23^ and were obtained from medical reports. It is possible for a record to have more than one of these diagnoses, as they are not mutually exclusive. The most common diagnosis is sleep-related breathing disorders (SRBD), which were diagnosed in 73% of all diagnostic nights, including obstructive sleep apnea (OSA), central sleep apnea, complex OSA and hypoventilation. Insomnia disorders were diagnosed in 16% of the cases and sleep-related movement disorders (SRMD) in 17%, including restless legs syndrome, periodic leg movement disorder (PLMND) and other SRMD. Parasomnia disorders were diagnosed in 13%, including rapid eye movement sleep behavior disorder (RBD) and non rapid eye movement parasomnias. Hypersomnia disorders were diagnosed in 5% of the cases including narcolepsy type 1 and narcolepsy type 2, Kleine-Levin syndrome and idiopathic hypersomnia. Normal findings, including no evidence of sleep disturbance or only mild SRBD (AHI < 15/h), were diagnosed in 8% of cases.

#### Sleep heart health study (SHHS) dataset

The Sleep Heart Health Study (SHHS)^15^ was a multicentre cohort study designed to investigate the association between sleep-disordered breathing and cardiovascular disease. The study consisted of two visits at which a PSG was obtained. Visit one (SHHS1) included 6441 participants between 1959 and 1998, and visit two (SHHS2) consisted of 3295 participants between 2001 and 2003. The study includes 5793 (SHHS1) and 2651 (SHHS2) full overnight PSGs performed at home.

#### Multi-ethnic study of atherosclerosis (MESA) dataset

The Multi-Ethnic Study of Atherosclerosis^17^ was investigating the factors associated with the development of subclinical cardiovascular disease and its progression to clinical disease in different ethnic groups. Of the 6814 participants, 2237 also underwent a sleep study including a full night of unattended PSG that took place between 2010 and 2012.

#### MrOS sleep study (MrOS) dataset

The MrOS Sleep Study^16^ aimed to investigate the relationship between sleep disorders and health outcomes such as falls, fractures and vascular disease in men aged 65 years and older. As part of the larger Osteoporotic Fractures in Men Study, which enrolled 5994 men, the Sleep Study subset included 3135 participants who underwent complete unattended polysomnography.

### Preprocessing

Like Olesen et al.^19^, we used three signals from the PSG for the arousal detection: the EEG to detect frequency shifts, the EOG as an indicator of R sleep and the chin EMG for the requirement of increased submental EMG in R sleep. During training, one channel was randomly selected for each signal to create a three-channel input.

We tested three different preprocessing steps from the literature^10^, using different sampling rates (50 Hz, 128 Hz and 200 Hz) and preprocessing steps. The method by Howe-Patterson et al.^9^ achieved the highest area under the precision-recall curve (AUPRC) on the MrOS validation set. We therefore kept this hyperparameter at 50 Hz during further optimization. An antialiasing finite impulse response (FIR) filter was applied to all signals before they were downsampled to 50 Hz. The signals are then normalised by removing the mean and root mean square over an 18 min moving window.

Based on the method used in the PhysioNet challenge 2018^18^, manual labelling was extended to include autonomous responses two seconds before the start and 10 s after the end of an arousal. The arousal labels were interpolated into a label signal so that the sampling rate matched the input signals and a sample wise evaluation is possible.

### Model architecture

We used a state-of-the-art model architecture described in the approach of Li and Guan^10^. We used an extensive grid search to optimize the architecture (number of layers, filter sizes, kernel size), choosing the best setting by best AUPRC on the DSDS validation set.

**Figure 2:**
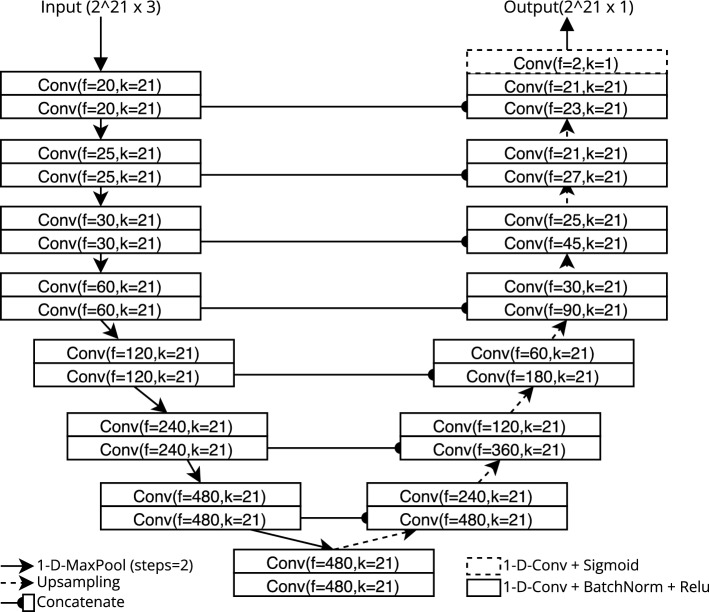
Final U-net architecture with eight layers of double convolution, where each convolution (Conv) uses a padding to preserve the length of the input, a number of filters (f) and a kernel size (k).

The final architecture is shown in Fig. 2 and uses fewer layers and a higher kernel size (k = 21) than the original model. Our final model takes the three-channel PSG signal with a sampling rate of 50 Hz as input. It produces a 50 Hz signal containing the probability of an arousal at any given time.

### Training

Prior to training, we performed a participant-wise train-test split of all datasets. We randomly selected participants for our holdout test sets, which resulted in 1202, 1698, 616 and 815 recordings for the DSDS, SHHS, MESA and MrOS datasets, respectively. The remaining data was divided into training (80% of participants) and validation (20% of participants) sets for each dataset.

We used the binary cross entropy as the a loss function and the Adam optimizer^24^ to update the weights of our model. To determine the learning rate (LR), we used an LR range test^25^. We cycled the LR between 0.003 and 0.00075 every four epochs. For regularisation, we randomly selected one of the available channels (e.g. right or left EOG) per signal type for each iteration. We also applied normalisation and added random noise with a factor between 0.8 and 1.3. If the validation metric (AUPRC) does not improve for 15 epochs, the training run stopped.

### Evaluation

For evaluation, we used metrics that are commonly used in the literature to compare our results at a sample and an event-based level. The most common metric for sample-based evaluation is the area under the precision-recall curve (AUPRC). For event and segment based evaluation the harmonic mean of precision and recall is used (F1 score).

To convert the predicted probabilities to events, we averaged our samples to a one second segment and used an approach from Brink-Kjaer et al.^27^. Where a threshold is used to determine positive samples, all consecutive positive samples are combined with a patience of ten seconds. Any arousal event under three seconds is discarded, as required by the AASM. We changed the minimum arousal length to 15 s to be consistent with our label manipulation and the patience to three seconds to be more precise. We optimized the threshold using the F1 score of the calculated events from the validation set. Each overlapping manual and automated scored event is marked as a true positive. The special case of two automated events overlapping a single manual event will result in one true positive and one false negative. Similarly, the case of two manual events overlapping an automated event will result in two true positives and one false positive. The final F1 score is calculated from the sum of the record-wise confusion matrices for the entire test set (micro F1 score).

To see how well our pre-trained model performed on data from other studies and scored by different experts, we also evaluated the model on different datasets using their specific test sets. We also wanted to see if a publicly available dataset would be suitable for scoring our clinical data. Therefore, we trained a model on each dataset-specific training set (e.g. *DSDS*_*test*_ trained on DSDS) and evaluated it on each hold-out test set (e.g. *SHHS1*_*test*_: scored on test data from SHHS1). None of the training or validation data was used for testing.

To understand the medical implications, we use the absolute difference between the predicted ArI and the ArI from the manual annotations on a per record basis and refer this as the ArI error. We tested whether our DSDS model had a bias in the ArI error for different patient age groups and sexes. We performed a Shapiro–Wilk test on the different groups, to see if the ArI error is normally distributed. Since the ArI error was not normally distributed between age groups and sexes, we proceeded by performing a Kruskal–Wallis test to investigate if there were significant differences between the groups. We performed a post-hoc Dunn test with Bonferroni correction to see which groups differ significantly.

We used the DSDS test set to assess the ability of public trained non-clinical data to detect sleep arousals in clinical data. We therefore grouped our test set into six different non-exclusive categories (without recordings using any SRBD related treatment) (see Table 3). We added 480 recordings to an exclusive category where SRBD was treated (including positive air presure therapy, positional therapy and mandibular advancement device therapy). This can provide insight into the model’s performance on recordings with therapy devices, which are generally excluded from public datasets^15–17^. We used Cohens D^26^ to calculate the effect size of the ArI error by comparing each category with the exclusive category normal findings. We tested each model to see if the effect size (ranging from none (0–0.2), small (0.2–0.5), moderate (0.5–0.8) and large (0.8–1) effect size) was different for each group.

**Table 3:** Sleep disorders categories within the test set.

Category	Included sleep disorder	Count
SRBD	obstructive sleep apnea (OSA), central sleep apnea, complex OSA, Hypoventilation	379
SRMD	restless legs syndrome, periodic leg movement disorder, other SRMD	121
Insomnia disorders	insomnia	161
Hypersomnia disorders	narcolepsy type 1/type 2, Kleine-Levin syndrome, chronic insomnia disorder	64
Parasomnia disorders	REM sleep behaviour disorder (RBD), Non rapid eye movement (NREM) parasomnias	116
normal finding	No evidence of sleep disorder or mild SRBD	100

SRBD: sleep-related breathing disorders, SRMD: sleep-related movement disorders, PLMD: periodic limb movement disorders.

### Ethics declarations

The use of the data for this research was approved by the local medical ethics committee (BO-EK-92032020) and allowed the retrospective use of the anonymous data without informed consent.

## Results

Based on ArI error the best results were achieved on the SHHS1 dataset with an AUPRC of 0.83, an F1 score of 0.80, an ArI correlation of 0.88 and an ArI error median of 1.71 (0.75–3.27), achieving state of the art results as shown in Table 1. The model trained and tested on our DSDS achieved an AUPRC of 0.71, an event-based F1 score of 0.74 and a Pearson correlation of 0.78 for arousal index prediction and an ArI error of 4.37 (1.88–7.72). The models trained on public datasets performed better than the model trained on our clinical DSDS, which may be due to the more heterogeneous test set.

### Evaluation across datasets

Evaluation across different datasets shows that each model performs best on its own test set for the technical metrics. This is shown for the F1 score in Table 4 and for the AUPRC in Table 5. The results seem to vary widely between datasets.

**Table 4:** F1 score for models separately trained and tested on the Dresden Sleep Dataset (DSDS), the first part of the Sleep Heart Health Study (SHHS1), the Multi-Ethnic Study of Atherosclerosis (MESA) and the MrOS Sleep Study (MrOS) datasets.

F1 Score	DSDS_test_	SHHS1_test_	MESA_test_	MrOS_test_
DSDS_train_	**0.74**	0.73	0.60	0.66
SHHS1_train_	0.71	**0.80**	0.71	0.74
MESA_train_	0.60	0.70	**0.81**	0.76
MrOS_train_	0.64	0.74	0.76	**0.80**

Bold values indicate the highest F1 score for each dataset.

**Table 5:** AUPRC score for models separately trained and tested on the Dresden Sleep Dataset (DSDS), the first part of the Sleep Heart Health Study (SHHS1), the Multi-Ethnic Study of Atherosclerosis (MESA) and the MrOS Sleep Study (MrOS) datasets.

AUPRC	DSDS_test_	SHHS1_test_	MESA_test_	MrOS_test_
DSDS_train_	**0.71**	0.70	0.50	0.58
SHHS1_train_	0.61	**0.83**	0.70	0.74
MESA_train_	0.50	0.71	**0.82**	0.77
MrOS_train_	0.55	0.75	0.76	**0.82**

Bold values indicate the highest AUPRC for each dataset.

### Medical implication

**Figure 3:**
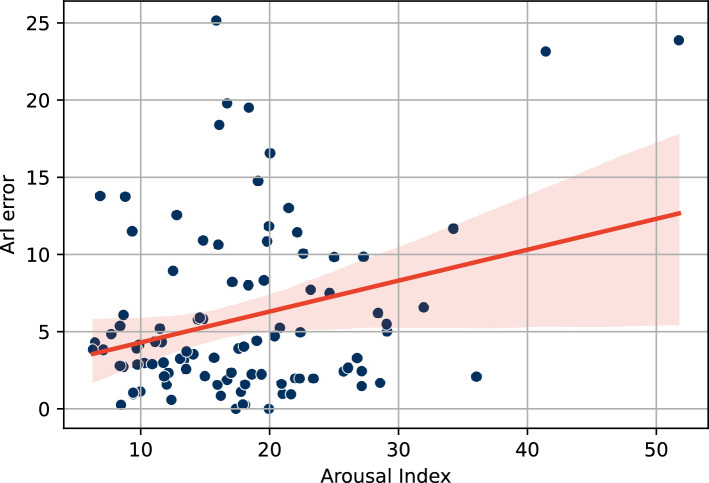
Correlation between Absolute difference in the arousal index (ArI error) and arousal index for the normal finding group in the Dresden Sleep Dataset (DSDS) using the model trained on DSDS.

The AUPRC and F1 Score metrics do not give a direct indication of ArI. Therefore, the ArI error was used to investigate the medical implications. It was found that the ArI error has a low, significant correlation (r = 0.28, *p* = 0.004) with the arousal index within the normal finding group, as shown in Fig. 3. This indicates that the number of prediction errors increases with the severity of the pathological changes (sleep fragmentation), regardless of the sleep disorder.

**Figure 4:**
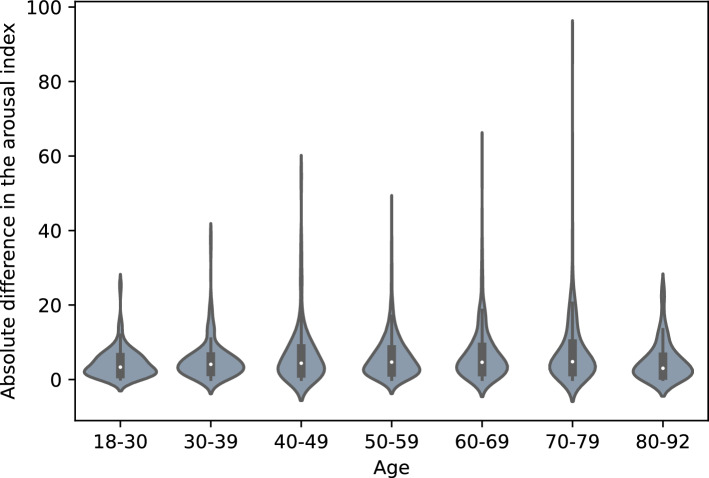
Influence of age on ArI error in the DSDS using the model trained on DSDS.

**Figure 5:**
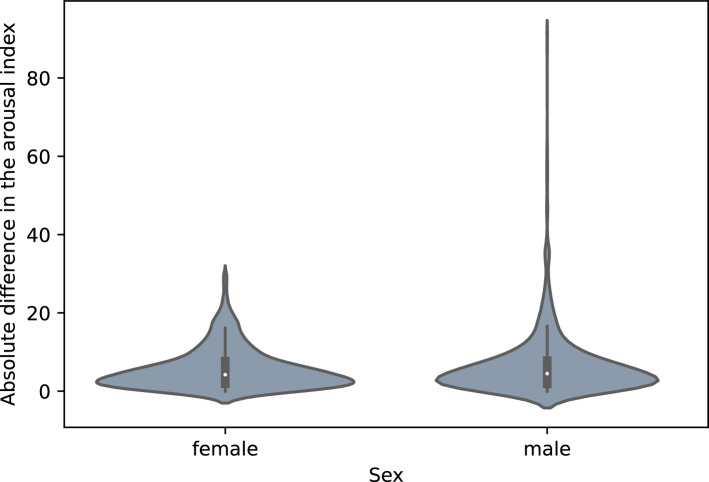
Influence of sex on ArI error in the DSDS using the model trained on DSDS.

Figure 4 shows the influence of age on the quality of the DSDS model using the ArI error. The Kruskal–Wallis test showed a significant difference in the age distribution, but the series of post-hoc Dunn tests revealed no significant differences between individual pairs of age groups at the 0.05 significance level. The Kruskal–Wallis test showed no significant difference in the ArI error for male or female patients, shown in Fig. 5.

**Figure 6:**
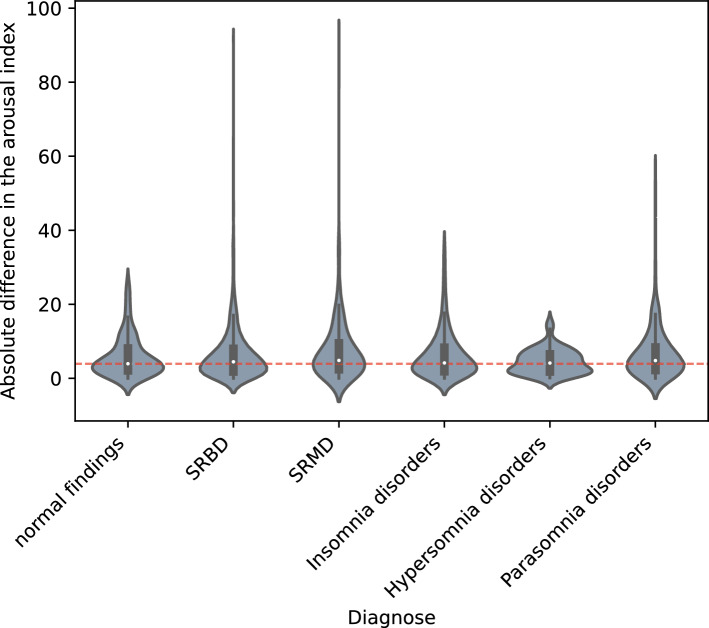
Influence of diagnosis on ArI error in the DSDS. The red dotted line indicates the median of the ArI error for the normal findings group.

The Fig. 6 shows slightly non-significant higher predicted ArI values for the SRBD and SRMD groups. Further details are provided in the next section, which shows the impact of the different training datasets.

### Medical implications of publicly trained models

The Table 6 shows that the ArI error showed similar variation between the datasets as the technical metrics (shown in Tables 4 and 5). Each model performs best on its own test set. The model trained on the SHHS1 dataset performed best on our clinical dataset, with only a slightly higher ArI error of 4.43 (2.00–8.91) compared to 4.37 (1.88–7.72) for the DSDS model.

**Table 6:** ArI error for models separately trained and tested on the Dresden Sleep Dataset (DSDS), the first part of the Sleep Heart Health Study (SHHS1), the Multi-Ethnic Study of Atherosclerosis (MESA) and the MrOS Sleep Study (MrOS) datasets.

ArI error	DSDS_test_	SHHS1_test_	MESA_test_	MrOS_test_
DSDS_train_	**4.37 (1.88–7.72)**	2.18 (1.01–4.20)	8.87 (4.35–14.21)	9.78 (5.57–16.63)
SHHS1_train_	4.43 (2.00–8.91)	**1.71 (0.75–3.27)**	4.45 (1.82–8.21)	6.49 (3.56–10.46)
MESA_train_	7.20 (3.49–12.85)	2.86 (1.33–5.10)	**3.20 (1.27–6.47)**	5.62 (2.84–9.70)
MrOS_train_	6.72 (2.93–12.56)	2.29 (0.99–4.57)	3.99 (1.75–7.14)	**3.43 (1.63–6.04)**

Bold values indicate the lowest ArI error for each dataset.

Finally, the performance of all models were evaluated on the DSDS test set. Clinical groups were grouped and compared to the normal findings group (as shown in Table 7). The model trained on the DSDS showed a small negative effect (Cohens D of 0.22 and 0.21) for the SRMD and SRBD groups and a small positive effect (Cohens D of − 0.3) for the hypersomnia disorder group. This positive effect was not observed in other models, with the model trained on the MESA dataset even showing a moderate negative effect (Cohens D of 0.72). Besides the hypersomnia disorder group, all public datasets showed a small to moderate negative effect on the other sleep disorders groups (Cohens D ranging from 0.2 to 0.73).

**Table 7:** Cohens D effect size for models trained on different datasets and evaluated on the Dresden Sleep Dataset (DSDS).

	DSDS_train_	SHHS1_train_	MESA_train_	MrOS_train_
SRBD	0.22	0.43*	0.63*	0.69*
SRBD-Therapy	− 0.09	0.29	0.36*	0.48*
SRMD	0.21	0.42	0.36	0.50*
Insomnia disorders	− 0.01	0.20	0.29	0.37
Hypersomnia disorders	− 0.30	0.18	0.72*	0.46
Parasomnia disorders	0.13	0.46*	0.66*	0.73*

Comparing the different clinical groups with the normal findings group with *indicating a significant difference (*p* < 0.05).

## Discussion

For the technical metrics F1 score and AUPRC, our models trained on publicly available datasets outperformed other approaches found in the literature (see Table 1). The model trained on the clinical DSDS still achieved state-of-the-art results, but was significantly lower than the other models. This may be due to the heterogeneous test set and the fact that we used data directly from the daily work of the sleep laboratory, without any re-evaluation or filtering for PSG quality. The technical metrics are well suited for comparing models at a low level of granularity, but may not be well suited for evaluating the actual task of detecting arousal events. Implementation details such as segment length, patience for combining events, and label manipulation as used in this work can affect these scores, making them difficult to compare with other approaches. Metrics based on the medical parameters of arousal index or arousal count give a direct insight into the impact of the algorithm when generating a sleep report. They are not influenced by implementation details. However, they negate false positives and negatives within recordings and may not be the best metrics to use during development.

In addition to the technical metrics, we chose to use the absolute difference in ArI for our evaluation because this metric is easy to interpret as it shows the expected difference in ArI during the night. A difference in the ArI, which can also be negative (as used in^7^), would negate the overestimation and underestimation of arousal events at the dataset level. It is therefore not suitable for comparing different clinical groups. A relative difference in the ArI or the F1 score would also be appropriate metrics. However, our model produces a negative correlation with these metrics and the ArI index within the normal findings group. This would lead to a bias in favour of healthy patients, as sleep disorders tend to have a higher ArI. The metric we used has a positive correlation with the ArI, which is also not ideal. However, this could explain the small negative effect on the SRBD and SRMD groups in our DSDS model, as these are the groups with the highest ArI.

Compared to other sleep-related scores, arousal scoring appears to have lower inter-rater agreement^5,28,29^. There is also a lack of large studies on inter-rater agreement in arousal detection. Other areas of sleep are better studied, such as respiratory events^30^. This makes it impossible to assess the quality of the current state of the art. The lower inter-rater agreement between experts also explains the lower results when transferring our model to another dataset.

With our model trained on the comprehensive clinical dataset, we have achieved state-of-the-art results with the model architecture and preprocessing we have optimized. Our model shows no age or sex bias and shows only small negative effects on some sleep disorders compared to healthy sleep. However, we also see some limitations in our DSDS. It is not known which record was scored by which expert. Due to the size of the dataset, we did not re-evaluate the records by another expert and used the data directly as a “real-World”-Dataset from the daily work of the sleep laboratory. Information on the ethnicity of our patients is also missing. The fact that we have multiple diagnoses for a single night may distort the evaluation of the clinical groups. In a larger test set, the evaluation could be done only on patients with an exclusive diagnosis.

The model, trained on a single dataset and tested on other datasets, shows a large shift in performance independent of sleep disorders. Approaches such as Brink-Kjaer et al.^27^, mix different datasets, which could have a positive impact^31^ on the diversity of experts and under-represented ethnicities. However, our aim was to demonstrate and compare current approaches using a dataset from a practising sleep laboratory. Therefore, we did not mix the datasets to show whether models with different data bases have a bias towards specific sleep disorders or SRBD therapy. However, mixing datasets may be an important approach for future work, especially when multiple clinical datasets are available.

The preprocessing by Howe-Patterson et al.^9^ gave the best results. By downsampling the signal to 50 Hz, only information up to 25 Hz is included, which is below the 35 Hz recommended by AASM. Although this may help to reduce the complexity for the model, it may also remove sleep-related information. A sampling rate of 70 Hz should be additionally investigated in the future.

Furthermore, we would like to point out that the transition from sample-wise to arousal event is taken from the literature^27^ and is not well investigated in our approach or in the literature in general. This could be a key factor in the performance evaluation of the models and should be further investigated.

## Conclusion

We have shown that it is possible to achieve state-of-the-art results on a large clinical dataset from the day-to-day work of a sleep laboratory. The performance of our model architecture trained and validated on one dataset varies when tested on different datasets. This needs to be considered when implementing automatic arousal detection for a sleep laboratory. In particular, when used to detect arousals in patients with sleep disorders. We propose that more approaches use additional metrics like the ArI error to evaluate their models, as they have medical relevance and show the expected error when used in practice.

Concluding from these results, a universally applicable model for automated arousal detection would require several clinical datasets from different centres and annotated sleep disorders. These should be mixed to reduce individual scorer bias. Other studies have shown that combining different micro-events during sleep improves results^21^. Therefore, we aim to further improve the results by adding other micro-events during sleep and sleep staging.

## Data Availability

The datasets SHHS, MESA and MrOS datasets are available from the National Sleep Research Resource website https://sleepdata.org/datasets. The DSDS data set used for the medical evaluation is available on reasonable request from the corresponding authors. The DSDS dataset is not publicly available because they contain information that could compromise the consent and privacy of research participants.

## Code availability

The complete implementation for the arousal detector was done using Python and Pytorch. The code and pretrained models are available on GitLab https://gitlab.com/sleep-is-all-you-need/arousaldetector under the GNU General Public License.
